# Association Between Pre-Admission ATRIA Scores and Initial Stroke Severity in Acute Ischemic Stroke: A Cross-Sectional Study

**DOI:** 10.3390/jcm14134665

**Published:** 2025-07-01

**Authors:** Hakan Süygün, Merve Akgül Günay, Damla Yalçınkaya Öner, Enes Çon, Mehmet Mustu, Ahmet Yılmaz, Sümeyye Fatma Ozer, Burçin Daş, Ahmet Karakurt, Özgür Akgul

**Affiliations:** 1 Department of Cardiology, Faculty of Medicine, Karamanoğlu Mehmetbey University, Karaman Training and Research Hospital, Üniversite Quarter 1984, Street. No. 1, 70200 Karaman, Turkey; enes_con@hotmail.com (E.Ç.); drmustumehmet@gmail.com (M.M.); dr.ahmetyilmaz@gmail.com (A.Y.); smyyftmzr@hotmail.com (S.F.O.); karakurt38@hotmail.com (A.K.); droakgul@yahoo.com (Ö.A.); 2 Department of Neurology, Faculty of Medicine, Karamanoğlu Mehmetbey University, Karaman Training and Research Hospital, 70200 Karaman, Turkey; mrv.akgl@hotmail.com; 3 Department of Cardiology, Karaman Training and Research Hospital, 70200 Karaman, Turkey; damlaykaya@gmail.com; 4 Department of Neurology, Torbalı State Hospital, 35860 İzmir, Turkey; bburcindas@gmail.com

**Keywords:** acute ischemic stroke, ATRIA score, atrial fibrillation, NIHSS score, stroke severity

## Abstract

**Objectives**: This study aimed to investigate the relationship between the anticoagulation and risk factors in atrial fibrillation (ATRIA) score and initial stroke severity in patients with acute ischemic stroke of varying etiologies, including atrial fibrillation (AF), large-artery atherosclerosis, and undetermined origin. **Methods**: In this prospective observational study, 416 patients admitted with acute ischemic stroke between June 2022 and December 2024 were analyzed. Stroke severity was assessed using the National Institutes of Health Stroke Scale (NIHSS), and patients were categorized into two groups: mild-to-moderate (NIHSS ≤ 15) and moderate-to-severe/severe (NIHSS > 15). Pre-admission ATRIA scores were calculated based on demographic and clinical parameters. Multivariable logistic regression was performed to assess the association between ATRIA scores and stroke severity. **Results**: Patients with more severe strokes had significantly higher ATRIA scores (median 8.5 vs. 5.0, *p* < 0.001). AF was more frequent in the severe group (44.8% vs. 31.3%, *p* = 0.037). In multivariable analysis, each one-point increase in the ATRIA score was independently associated with a 1.82-fold increase in the odds of severe stroke (OR 1.823, 95% CI 1.568–2.119, *p* < 0.001). High ATRIA scores (>6) were associated with an 11.7-fold increased risk of severe stroke (OR 11.692, 95% CI 5.636–24.255, *p* < 0.001), independent of stroke etiology, ejection fraction, and inflammatory markers. **Conclusions**: The ATRIA score is independently associated with initial stroke severity across diverse ischemic stroke etiologies. It may serve as a simple, practical tool for early risk stratification in the acute setting, regardless of AF status. Further studies are warranted to confirm its utility in guiding early management and prognosis.

## 1. Introduction

Stroke remains a leading cause of disability and mortality worldwide, with a wide spectrum of clinical severity influenced by multiple factors, including etiology and baseline comorbidities [1]. The initial severity of stroke has a direct impact on patient outcomes, including early mortality, length of hospitalization, and long-term functional recovery [2]. As such, early identification of patients likely to present with severe strokes is critical in emergency care and resource allocation.

Atrial fibrillation (AF) represents one of the most clinically significant cardiac arrhythmias and serves as a major independent risk factor for ischemic stroke, contributing to approximately 15–20% of all cerebrovascular events and worse functional outcomes compared to other stroke subtypes [3,4,5]. To estimate thromboembolic risk and guide anticoagulation in AF patients, clinical risk scores such as CHADS_2_ (Congestive heart failure, Hypertension, Age ≥ 75, Diabetes mellitus, and prior Stroke or TIA) and CHA_2_DS_2_-VASc (Congestive heart failure, Hypertension, Age ≥ 75 [2 points], Diabetes mellitus, prior Stroke or TIA [2 points], Vascular disease, Age 65–74, and female Sex category) are widely used [6,7]. Although these scores were originally developed for stroke prevention, subsequent research has demonstrated moderate associations between higher CHADS_2_ and CHA_2_DS_2_-VASc scores and greater initial stroke severity, as measured by the National Institutes of Health Stroke Scale (NIHSS), in both AF and non-AF populations [8,9,10,11,12,13].

More recently, the anticoagulation and risk factors in atrial fibrillation (ATRIA) score has emerged as an alternative tool, incorporating additional variables such as renal dysfunction and proteinuria to improve stroke risk prediction [14]. However, unlike the more established CHADS_2_ and CHA_2_DS_2_-VASc scores, data regarding the ATRIA score’s association with stroke severity remain limited, especially in heterogeneous patient populations that include large-artery atherosclerosis, AF, and strokes of undetermined etiology.

The NIHSS is a widely used tool for objectively evaluating stroke severity at admission and predicting long-term outcomes [15]. Due to its strong correlation with both short- and long-term clinical outcomes, the NIHSS serves as an ideal metric for assessing potential associations with risk prediction scores [2]. Given its prognostic value, the NIHSS serves as a robust benchmark for assessing the potential of pre-admission risk scores to reflect stroke severity. While existing evidence supports a relationship between CHADS_2_/CHA_2_DS_2_-VASc and NIHSS, the predictive performance of the ATRIA score in this context remains underexplored. Considering the ease of calculating the ATRIA score from routine clinical data and its ability to reflect systemic vascular burden and cardio-renal comorbidities, the score may serve as a valuable triage tool in acute stroke settings. Its potential to predict initial stroke severity across diverse stroke etiologies could facilitate earlier identification of high-risk patients, guide urgent decision-making, and optimize resource allocation.

In this study, we investigated the association between pre-admission ATRIA scores and stroke severity assessed by NIHSS in all acute ischemic stroke patients. Through additional subgroup analyses, we further explored the robustness of this relationship across diverse stroke etiologies, thereby expanding the potential utility of the ATRIA score as a rapid, accessible prognostic tool in the acute stroke setting.

## 2. Methods

### 2.1. Patient Population and Study Design

This cross-sectional, observational study was conducted in the emergency department of Karaman Training and Research Hospital between June 2022 and December 2024. All initial evaluations, including clinical, laboratory, and radiological assessments, were completed in this setting. A total of 580 consecutive patients admitted with ischemic stroke were initially screened.

**Figure 1:**
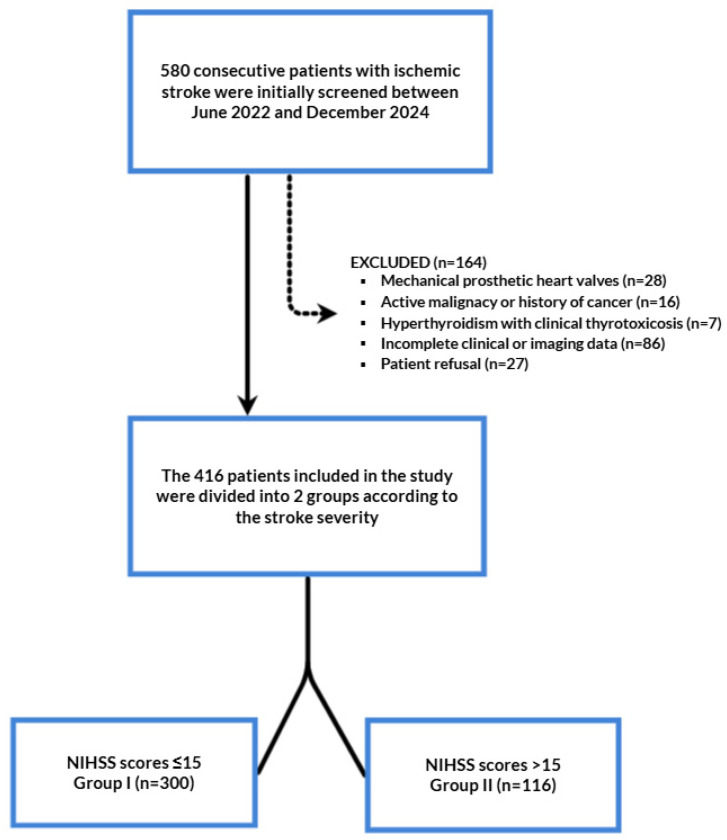
Flow chart of the study. NIHSS: National Institutes of Health Stroke Scale.

All patients presenting with symptoms suggestive of acute stroke were assessed under a standardized stroke alert protocol (‘code stroke’). Upon emergency department arrival, the multidisciplinary stroke team—including neurologists, emergency physicians, and radiologists—was immediately notified. Neurological assessment with NIHSS scoring, vital sign stabilization, laboratory testing, and neuroimaging were performed within the first 60 min following hospital presentation [16]. The diagnosis of ischemic stroke was confirmed by experienced neurologists based on clinical evaluation and cranial magnetic resonance imaging (MRI) findings. For descriptive purposes, stroke severity was also classified into four NIHSS categories: minor (NIHSS 0–4), moderate (NIHSS 5–15), moderate-to-severe (NIHSS 16–20), and severe (NIHSS 21–42), in accordance with widely used clinical stratification schemes [2,17]. Stroke etiology was classified according to Trial of Org 10172 in Acute Stroke Treatment (TOAST) criteria, and all etiologic subtypes were included in the analysis (AF, large-artery atherosclerosis, and undetermined origin) [2]. Patients were divided into two groups based on their initial NIHSS scores using the established cutoff of ≥15, which indicates severe stroke with poor short-term prognosis, to enable clinically meaningful outcome analysis [2,17,18,19]. Patients with NIHSS scores ≤ 15 were included in group I (mild-to-moderate), while those with NIHSS scores > 15 were included in group II (moderate-to-severe and severe). Exclusion criteria were defined as the following; mechanical prosthetic heart valves, active malignancy or history of cancer, hyperthyroidism (TSH < 0.1 mIU/L with clinical thyrotoxicosis), incomplete clinical/imaging data, and patient refusal. Following the application of exclusion criteria, a total of 416 patients were examined (Figure 1). Our study complied with the Declaration of Helsinki and was approved by the local ethical committee of the Karamanoglu Mehmetbey University, Karaman, Turkey, and written informed consent was obtained from all participants or their legal representatives.

### 2.2. Stroke Etiology

All patients underwent mandatory 24-h Holter monitoring as part of cardiac evaluation. Cases with undetermined stroke etiology received extended monitoring (up to 72 h), supplemented by continuous telemetry during hospitalization. Vascular evaluation included comprehensive extracranial Doppler ultrasonography, supplemented by computed tomography angiography for intracranial assessment when clinically warranted. All patients additionally underwent echocardiography (transthoracic or transesophageal based on clinical suspicion). For etiological classification, patients were categorized through a dynamic process that initially identified AF-associated strokes (including both pre-existing and incident AF detected during follow-up), large artery atherosclerosis (defined by carotid or vertebral stenosis ≥ 50%), and cases of undetermined origin after complete evaluation. Notably, patients initially classified as undetermined etiology who subsequently developed AF during the follow-up (detected through systematic monitoring including serial Holter examinations and event-triggered ECG recording) were reclassified into the AF group. This classification approach ensured accurate etiological attribution while accounting for the dynamic nature of arrhythmia detection in stroke patients. Although extended follow-up was conducted to refine stroke etiology, this process was not part of the core research objective, and both ATRIA and NIHSS scores were assessed concurrently at baseline.

### 2.3. ATRIA Score

The ATRIA stroke risk score was calculated for all patients by assigning weighted points to five clinical parameters: advanced age (0–9 points based on age brackets and prior stroke history), female sex (1 point), diabetes mellitus (DM) (1 point), hypertension (HT) (1 point), and renal dysfunction (1 point for proteinuria or eGFR < 45 mL/min/1.73 m^2^) (Supplementary Table S1). The total score was categorized into three risk strata according to the original ATRIA study: 0–5 points (low annual stroke risk, <1%), 6 points (intermediate risk, 1–2%), and >6 points (high risk, >2%) [14]. For analysis, we calculated pre-admission ATRIA scores using documented medical history and admission laboratory values, with renal function assessed through serum creatinine-based eGFR (CKD-EPI equation) and urinalysis for proteinuria.

### 2.4. Statistical Analysis

The data were analyzed using IBM SPSS Statistics ver. 25 (IBM Corporation, Armonk, NY, USA) package program. The Kolmogorov–Smirnov test was employed to ascertain the normality of the distribution of continuous variables, and Levene’s test was utilized to determine the fulfillment of the assumption of homogeneity of variances. Categorical data were expressed as either numbers (*n*) and percentages (%). Quantitative data were given as the mean ± standard deviation (SD) or the median (25th–75th percentile), as appropriate. The mean differences between groups were compared by Student’s *t* test, while the Mann–Whitney U test was applied for comparisons of non-normal data. The analysis of qualitative data was conducted using Pearson’s χ^2^ test. Following the adjustment for confounding factors, the effect of ATRIA scores on the discrimination between patients with low-to-moderate stroke and those with moderate-to-severe or severe strokes was investigated by means of multiple logistic regression analysis. Multivariate analyses were conducted through two separate models including ATRIA score as a continuous variable in model 1 and ATRIA score as a categorical variable in model 2. Variables with univariable tests that yielded *p* values less than 0.10 were considered for inclusion in the multivariable models. Odds ratios and 95% confidence intervals for each independent variable were also calculated. The statistical significance of the results was determined by a *p*-value of 0.05. Spearman’s rank correlation test was used to assess the relationship between ATRIA and NIHSS scores. Correlation coefficients were interpreted as follows: 0.00–0.19 = very weak, 0.20–0.39 = weak, 0.40–0.59 = moderate, 0.60–0.79 = strong, and 0.80–1.0 = very strong correlation [20]. Additionally, to evaluate the relationship between ATRIA scores and different stroke etiologies, we compared the distribution of ATRIA scores across stroke subtypes classified by TOAST criteria. These comparisons are presented in Supplementary Table S2. Forest plots were generated in JASP v0.19.3.0 exclusively to provide a visual comparison of the ATRIA score’s ability to predict severe stroke within each etiological subgroup.

## 3. Results

After applying inclusion criteria, 416 patients were included in the final analysis. The mean age was 71.8 ± 10.2 years (range 37–96), and 200 (48.1%) were female. Stroke etiology was undetermined in 143 (34.4%) patients, attributed to carotid artery disease in 123 (29.5%), and AF in 150 (36.1%). The median ATRIA score was 6 in total population; 164 patients (39.4%) had low (0–5), 73 (17.5%) had intermediate (6), and 179 (43.0%) had high (>6) ATRIA scores. The overall median NIHSS at admission was 9; 126 patients (30.3%) were classified as minor (NIHSS 0–4), 174 (41.8%) as moderate (NIHSS 5–15), 72 (17.3%) as moderate-to-severe (NIHSS 16–20), and 44 (10.6%) as severe (NIHSS 21–42) (Table 1).

For subsequent analyses, patients with NIHSS ≤ 15 (minor + moderate; *n* = 300) were categorized as group I, while those with NIHSS > 15 (moderate-to-severe + severe; *n* = 116) were classified as group II. Comparison of group I and group II (Table 2) showed no significant differences in body mass index, smoking history, history of coronary artery disease, white blood cell count, or lipid profile (*p* > 0.05). In contrast, group II patients were older (74.6 ± 9.1 vs. 70.7 ± 10.5 years, *p* < 0.001), more often female (55.2% vs. 44.0%, *p* = 0.032), and had higher prevalences of DM (48.3% vs. 31.0%, *p* = 0.001), chronic heart failure (22.4% vs. 11.7%, *p* = 0.004), HT (84.5% vs. 69.3%, *p* = 0.001), proteinuria (29.3% vs. 14.0%, *p* < 0.001), reduced glomerular filtration rate (<60 mL/min/1.73 m^2^; 47.4% vs. 24.7%, *p* < 0.001), and prior stroke or transient ischemic attack (38.8% vs. 21.3%, *p* < 0.001). They also had significantly higher median ATRIA scores (8.5 [7,8,9,10] vs. 5 [4,5,6,7], *p* < 0.001), larger left atrial diameters, higher HbA_1_c, and elevated C-reactive protein (CRP), but lower left ventricular ejection fraction (EF), thyroid-stimulating hormone (TSH), hemoglobin, and albumin levels (all *p* < 0.05). AF was more frequent in group II (44.8% vs. 31.3%, *p* = 0.037), while undetermined strokes were less frequent (20.7% vs. 40.3%, *p* < 0.001). The difference in carotid artery etiology did not reach statistical significance (34.5% vs. 27.3%, *p* = 0.065).

**Figure 2:**
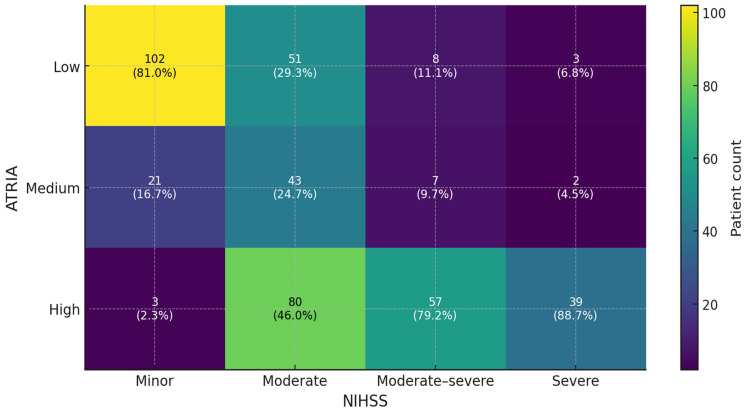
The heat map of the frequency distribution of ATRIA scores of the patients according to mild (1–4), moderate (5–15), moderate-to-severe (16–20), and severe (21–42) stroke severity groups in terms of NIHSS. ATRIA: anticoagulation and risk factors in atrial fibrillation; NIHSS: National Institutes of Health Stroke Scale.

**Figure 3:**
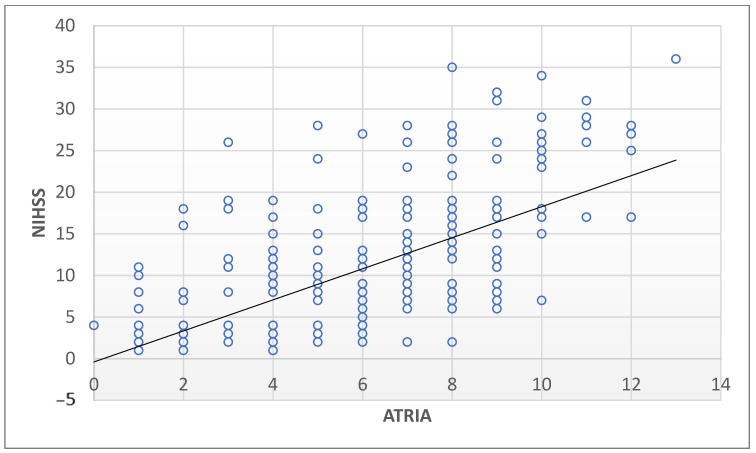
Scatter plot showing the correlation between ATRIA and NIHSS scores with regression line. ATRIA: anticoagulation and risk factors in atrial fibrillation, NIHSS: National institutes of Health Stroke Scale.

The distribution of ATRIA score categories differed significantly between groups (*p* < 0.001): low- and intermediate-risk ATRIA scores predominated in group I, while high-risk scores were significantly more prevalent in group II (NIHSS > 15). Figure 2 presents a heat map of the frequency distributions of ATRIA scores of the patients according to mild (1–4), moderate (5–15,) moderate-to-severe (16–20), and severe (21–42) stroke severity groups in terms of NIHSS. Moreover, there was a strong positive correlation between ATRIA and NIHSS scores (Spearman’s r = 0.641, *p* < 0.001) (Figure 3).

In multivariable logistic regression (Table 3), each one-point increase in ATRIA score as a continuous variable in model 1 was independently associated with a 1.82-fold increase in the odds of being in group II (severe-to-very-severe stroke; OR 1.823, 95% CI 1.568–2.119, *p* < 0.001), after adjustment for CRP (OR 1.009 per mg/L, 95% CI 1.001–1.017, *p* = 0.021), stroke etiology (carotid vs. undetermined: OR 2.190, 95% CI 1.078–4.447, *p* = 0.030), and EF (OR 0.964 per %, 95% CI 0.932–0.997, *p* = 0.031). When the ATRIA score was analyzed as a categorical variable in model 2 (Table 3), patients with high ATRIA scores (>6) exhibited a markedly increased likelihood of presenting with severe-to-very-severe stroke, with an odds ratio of 11.692 (95% CI: 5.636–24.255, *p* < 0.001). In the multivariable models, reduced left ventricular EF (OR: 0.958 per %, 95% CI: 0.928–0.989, *p* = 0.008), AF etiology (OR: 1.578, 95% CI: 1.273–1.863, *p* = 0.021), carotid artery etiology (OR: 2.552, 95% CI: 1.268–5.135, *p* = 0.009), and elevated CRP levels (OR: 1.009 per mg/L, 95% CI: 1.001–1.016, *p* = 0.025) also remained independently associated with increased stroke severity.

Subgroup analyses by stroke etiology revealed that the association between higher ATRIA scores and increased stroke severity (NIHSS ≥ 15) remained consistent across all etiological categories. In patients with cryptogenic stroke, each one-point increase in ATRIA score independently increased the odds of presenting with moderate-to-severe stroke by 1.67 (OR = 1.671, 95% CI: 1.270–2.197, *p* < 0.001). In the large-artery atherosclerosis subgroup (carotid etiology), a high ATRIA score was significantly associated with severe stroke risk (OR = 2.217, 95% CI: 1.583–3.105, *p* < 0.001), along with reduced EF (OR = 0.900, 95% CI: 0.821–0.986, *p* = 0.024).

Among patients with AF-related stroke, the predictors of severe stroke (NIHSS ≥ 15) included high ATRIA score (OR = 1.712, 95% CI: 1.348–2.173, *p* < 0.001), reduced EF (OR = 0.957, 95% CI: 0.918–0.999, *p* = 0.044), and elevated CRP levels (OR = 1.022, 95% CI: 1.007–1.037, *p* = 0.004).

**Figure 4:**
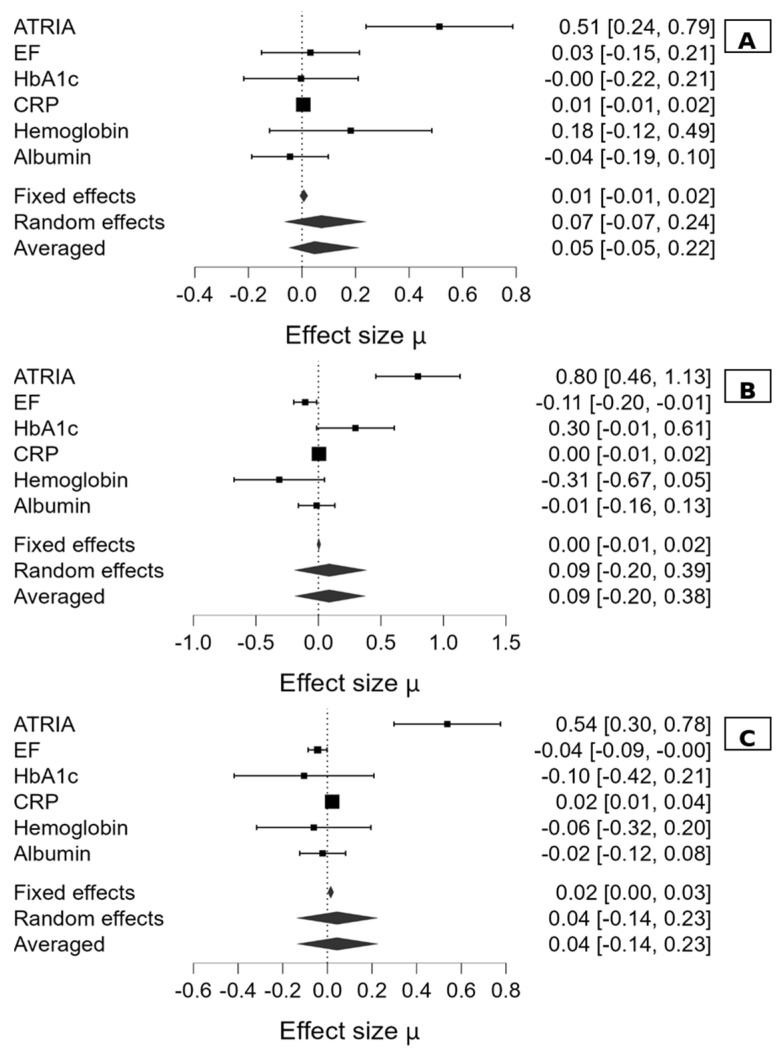
Subgroup forest plots demonstrating the association between candidate factors and stroke severity (NIHSS ≥ 15) across etiologic subgroups: (**A**) Cryptogenic stroke, (**B**) Carotid artery disease-related stroke, and (**C**) Atrial fibrillation-related stroke. ATRIA: anticoagulation and risk factors in atrial fibrillation score, EF: ejection fraction, CRP: C-reactive protein. Each square represents the regression-based effect size (standardized β coefficient, μ), with horizontal lines showing 95% confidence intervals (CI); square area is proportional to the inverse of variance. Note: These effect sizes are not odds ratios but standardized β coefficients from multivariable logistic regression models. Diamonds indicate fixed-effect, random-effect, and averaged estimates. CI bars not crossing zero reflect statistically significant associations.

These consistent findings across etiological subtypes suggest the robustness of the ATRIA score as a predictor of stroke severity, regardless of underlying stroke mechanism (Figure 4).

## 4. Discussion

This study demonstrates an independent association between pre-admission ATRIA scores and initial stroke severity, as measured by NIHSS, in a heterogeneous group of acute ischemic stroke patients. To our knowledge, this current study is the first to examine the relationship between the ATRIA score and stroke severity in the general ischemic stroke population regardless of AF status or treatment modality. It provides new insights into the ATRIA score’s broader applicability in assessing stroke severity across different etiologies.

CHADS_2_ and CHA_2_DS_2_-VASc are well-validated scores to predict stroke risk, stroke severity, and functional outcomes, both within AF-related and non-AF-related stroke patients [10,21,22]. However, since CHADS_2_ and CHA_2_DS_2_-VASc scores have only a moderate ability to predict stroke risk in patients with atrial fibrillation [23], the ATRIA score was developed to provide more accurate and reliable risk stratification. It was demonstrated that the ATRIA score was superior in predicting stroke risk and it identified a significantly larger proportion of patients at low stroke risk [14]. Besides the stroke risk, it also predicted stroke-related severe adverse clinical outcomes. However, limited data exist on the relationship between the ATRIA score and acute stroke severity, especially in heterogeneous populations including AF, large-artery atherosclerosis, and strokes of undetermined or other etiologies. The study by Kim et al. similarly evaluated the ATRIA score in acute ischemic stroke patients undergoing endovascular thrombectomy and found a positive correlation with NIHSS scores. Our study extends these findings to a broader population of stroke patients, irrespective of treatment modality or AF status [24]. Our findings extend prior knowledge by revealing that the ATRIA score correlates strongly with NIHSS scores, regardless of the underlying stroke mechanism and treatment modality. Patients with higher ATRIA scores were more likely to present with severe strokes (NIHSS > 15) independently, even after adjusting for confounding factors such as CRP, EF, and stroke etiology.

The fact that ATRIA scores’ predictive value irrespective of the etiology shows us that ATRIA components capture systemic conditions that predispose patients to more extensive brain injury. For example, advanced age, renal impairment, and HT are known to promote atherosclerosis, endothelial dysfunction, and pro-inflammatory states, all of which can contribute to larger infarcts and worse neurological deficits [25,26]. Small vessel disease has an important role in cerebrovascular disease and is a leading cause of cognitive decline and functional loss. HT, increasing age, DM, and renal impairment are known to exacerbate cerebral small vessel disease and increase susceptibility to ischemic injury [27,28,29]. As expected, all the above-mentioned comorbidities were more prevalent in those with an NIHSS > 15. In addition to score parameters, inflammation plays a major role in atherothrombosis, and elevated markers such as CRP have been linked to larger infarct volume and worse prognosis [30]. In our study, elevated CRP levels in patients with high ATRIA scores suggest a pro-inflammatory state that may increase ischemic damage.

Our multivariable analysis revealed a significant association between carotid artery etiology and higher NIHSS scores, indicating greater stroke severity. This finding correlates with the existing literature demonstrating that carotid atherosclerotic strokes often present with more severe neurological deficits, likely due to the larger infarct sizes resulting from critical stenosis or occlusion of major extracranial cerebral arteries [31,32]. This is not surprising since patients with carotid artery disease are older and have more atherosclerotic risk factors, including DM, smoking, HT, and high cholesterol [33]. Renal impairment was also found to be associated with carotid artery disease. A previous study of patients with no previous history of kidney failure demonstrated that renal function was inversely associated with carotid atherosclerosis, independent of other well-established risk factors such as age, gender, blood pressure, serum LDL cholesterol, and hyperglycemia [34]. The presence of multiple comorbidities such as HT, DM, and renal dysfunction, which are also represented in the ATRIA score, increases vascular pathology and may predispose an individual to more extensive ischemic damage due to impaired collateral circulation and microvascular vulnerability [35,36]. Considering the correlation of the ATRIA score with stroke severity, the increased severity of stroke attributable to carotid artery disease, driven by overlapping risk factors, is not surprising.

In addition to its role as a common etiological factor in ischemic stroke, AF appears to influence the initial clinical presentation by contributing to more severe strokes. AF was significantly more prevalent in patients with severe-to-very-severe stroke (group II), consistent with the well-established association between AF and more disabling ischemic strokes. This finding supports previous studies showing that AF-related strokes tend to be more severe at presentation, potentially due to larger embolic infarcts and limited collateral circulation [3,5]. In our study, even after adjusting for confounding variables such as EF and CRP, AF remained independently associated with higher NIHSS scores. This reinforces the notion that AF itself, beyond its role as a stroke trigger, may reflect an underlying prothrombotic and cardioembolic state contributing to more extensive brain injury.

We found that left atrial enlargement and reduced EF were significantly higher in group II. Emerging evidence suggests that atrial dysfunction—including atrial cardiopathy and left atrial enlargement—may serve as a marker of thromboembolic potential even in the absence of clinically apparent AF [37,38]. Left-atrial enlargement, which was significantly more prominent in patients with higher ATRIA scores in our cohort, has been independently associated with both greater stroke severity and worse outcomes [39]. The concept of atrial cardiomyopathy, encompassing structural and electrical abnormalities of the atria, is increasingly recognized as a distinct pathophysiological substrate for embolic stroke [40].

Our subgroup analyses further strengthen the clinical relevance of the ATRIA score by demonstrating its consistent predictive value across different stroke etiologies. Regardless of whether the underlying cause was AF, carotid artery disease, or cryptogenic, elevated ATRIA scores were significantly associated with moderate-to-severe strokes (NIHSS ≥ 15). This observation suggests that the ATRIA score reflects a broader systemic risk profile that transcends the stroke mechanism and may be useful in the early triage and risk stratification of diverse ischemic stroke patients. These findings are visualized in our forest plot (Figure 4), reinforcing the robustness and potential generalizability of ATRIA as a surrogate marker of stroke severity.

From a clinical perspective, our findings highlight the potential utility of the ATRIA score as a simple, non-invasive tool for early stroke severity stratification across a broad spectrum of ischemic stroke patients. Easily calculable using routine admission data, the ATRIA score may reflect systemic vascular burden and cardio-renal comorbidities that predispose patients to more severe strokes. In the emergency department setting, patients presenting with elevated ATRIA scores could be prioritized for intensive neurological monitoring, urgent neuroimaging, and early involvement of stroke teams. This may improve triage efficiency, particularly in time-sensitive situations. Notably, the score’s predictive value was observed irrespective of AF status or stroke etiology, suggesting broader applicability beyond its original use in guiding anticoagulation decisions. In resource-limited settings, where advanced diagnostics or continuous monitoring may not be feasible, the ATRIA score could serve as a bedside marker to identify patients at higher risk for adverse neurological outcomes. Thus, it may support early intervention strategies, guide resource allocation, and complement existing stroke severity scales in acute care planning.

This study has several limitations that warrant consideration. First, it was conducted at a single tertiary care center, which may limit the generalizability of our findings to broader populations. Second, although we used strict criteria for etiological classification and performed extended cardiac monitoring, subclinical AF or other occult causes of stroke may still have gone undetected. Third, while the NIHSS provides a robust assessment of neurological severity, it does not capture infarct volume or topographic patterns, which may also influence functional outcomes. Finally, this was an observational study, and although multivariable analyses were performed, causality cannot be definitively established. In our multivariable analysis, covariates were selected based on univariate *p*-values < 0.10. While this approach is widely used, we acknowledge that it may introduce selection bias and limit causal inference.

## 5. Conclusions

In conclusion, our findings demonstrate that higher pre-admission ATRIA scores are significantly associated with greater initial stroke severity, as measured by the NIHSS, in a diverse population of patients with acute ischemic stroke. This relationship persisted across multiple etiologic subgroups and remained robust after adjusting for key clinical variables. The ATRIA score, originally developed to estimate thromboembolic risk in patients with AF, appears to also reflect systemic vascular burden and organ dysfunction that contribute to more severe neurological injury. Given its simplicity, accessibility, and strong correlation with stroke severity, the ATRIA score may serve as a practical adjunct for early risk stratification and clinical decision-making in the acute stroke setting. Future prospective, multicenter studies are warranted to validate its prognostic utility and explore its role in guiding personalized management strategies in acute cerebrovascular care.

## Figures and Tables

**Table 1:** Demographic and clinical characteristics of all cases.

	*n* = 416
A. Demographics	
Age	71.8 ± 10.2
Female sex	200 (48.1%)
BMI (kg/m^2^)	25.2 ± 2.0
Smoking	115 (27.6%)
B. Comorbidities	
Hypertension	330 (79.3%)
Diabetes mellitus	204 (49.0%)
Chronic heart failure	71 (17.1%)
Prior stroke or TIA	48 (11.5%)
Proteinuria	113 (27.2%)
Low eGFR (<60 mL/min/1.73 m^2^)	43 (10.3%)
Coronary artery disease	90 (21.6%)
C. Stroke Etiology (TOAST classification)	
Atrial fibrillation	150 (36.1%)
Carotid artery disease	123 (29.5%)
Undetermined etiology	143 (34.4%)
D. ATRIA and NIHSS Scores	
ATRIA score	6.0 (4.0–8.0)
ATRIA risk category	
Low (0–5)	164 (39.4%)
Intermediate (6)	73 (17.5%)
High (>6)	179 (43.0%)
NIHSS score	9.0 (4.0–17.0)
Minor (0–4)	126 (30.3%)
Moderate (5–15)	174 (41.8%)
Moderate-to-severe (16–20)	72 (17.3%)
Severe (21–42)	44 (10.6%)
E. Laboratory Parameters	
HbA1c (%)	6.2 (5.8–7.9)
CRP (mg/L)	6.8 (2.7–24.2)
Albumin (g/L)	38.0 (34.0–41.0)
Hemoglobin (g/dL)	13.3 ± 1.93
WBC (10^3^/μL)	8.6 (6.9–10.6)
Total cholesterol (mg/dL)	180.0 (150.0–205.0)
LDL cholesterol (mg/dL)	106.5 (86.0–129.5)
HDL cholesterol (mg/dL)	44.8 ± 9.1
TSH (μIU/mL)	1.4 (0.9–2.3)
EF (%)	55.0 (55.0–60.0)
LA diameter (cm)	3.7 (3.5–4.4)

BMI: body mass index, CAD: coronary artery disease, CHF: chronic heart failure, CRP: C- reactive protein, DM: diabetes mellitus, EF: ejection fraction, GFR: glomerular filtration rate, HDL: high-density lipoprotein, HT: hypertension, LA: left atrium, LDL: low-density lipoprotein, TIA: transient ischemic attack, TSH: thyroid-stimulating hormone, WBC: white blood cell. Descriptive statistics for continuous variables are shown as mean ± SD or median (25th–75th) percentiles, where appropriate.

**Table 2:** Demographic and clinical characteristics of patients according to groups in terms of NIHSS.

	Group I (*n* = 300)	Group II (*n* = 116)	*p*-Value
Demographics			
Age (years)	70.0 ± 9.8	76.3 ± 9.6	<0.001 ^A^
Gender			0.007 ^B^
Female	132 (44.0%)	68 (58.6%)	
Male	168 (56.0%)	48 (41.4%)	
BMI (kg/m^2^)	25.3 ± 2.1	25.0 ± 1.9	0.255 ^A^
Smoking	86 (28.7%)	29 (25.0%)	0.453 ^B^
Comorbidities			
DM	137 (45.7%)	67 (57.8%)	0.027 ^B^
CHF	44 (14.7%)	27 (23.3%)	0.036 ^B^
HT	222 (74.0%)	108 (93.1%)	<0.001 ^B^
Proteinuria	51 (17.0%)	62 (53.4%)	<0.001 ^B^
Low GFR	20 (6.7%)	23 (19.8%)	<0.001 ^B^
Prior stroke or TIA	16 (5.3%)	32 (27.6%)	<0.001 ^B^
CAD	63 (21.0%)	27 (23.3%)	0.613 ^B^
Stroke Etiology (TOAST classification)			<0.001 ^B^
Undetermined	120 (40.0%)	23 (19.8%)	
Carotid artery disease	81 (27.0%)	42 (36.2%)	
Atrial fibrillation	99 (33.0%)	51 (44.0%)	
ATRIA			
ATRIA	5.0 (4.0–7.0)	8.5 (7.0–10.0)	<0.001 ^C^
ATRIA score category			<0.001 ^B^
Low (0–5)	153 (51.0%)	11 (9.5%)	
Intermediate (6)	64 (21.3%)	9 (7.8%)	
High (7–15)	83 (27.7%)	96 (82.8%)	
Laboratory Parameters			
EF (%)	60.0 (55.0–60.0)	55.0 (50.0–55.0)	<0.001 ^C^
LA diameter (cm)	3.7 (3.5–4.3)	4.0 (3.7–4.5)	<0.001 ^C^
HbA1c (%)	6.2 (5.7–7.7)	6.6 (5.9–8.5)	0.021 ^C^
CRP (mg/L)	5.5 (2.0–12.0)	22.0 (6.4–48.4)	<0.001 ^C^
TSH (μIU/mL)	1.4 (1.0–2.3)	1.2 (0.8–2.1)	0.034 ^C^
Hemoglobin (g/dL)	13.5 ± 1.98	12.9 ± 1.73	0.002 ^A^
WBC (10^3^/μL)	8.6 (6.9–10.5)	9.1 (6.7–11.0)	0.484 ^C^
Albumin (g/L)	39.0 (35.4–41.0)	35.0 (32.0–39.6)	<0.001 ^C^
Total cholesterol (mg/dL)	181.5 (151.3–205.0)	172.0 (146.5–200.5)	0.251 ^C^
LDL cholesterol (mg/dL)	109.5 (89.0–129.9)	102.0 (81.0–128.8)	0.106 ^C^
HDL cholesterol (mg/dL)	44.5 ± 9.3	45.6 ± 8.6	0.292 ^A^

Cases classified as minor or moderate stroke were assigned to Group I, whereas those categorized as moderate-to-severe or severe stroke were assigned to Group II. BMI: body mass index, CAD: coronary artery disease, CHF: chronic heart failure, CRP: C- reactive protein, DM: diabetes mellitus, EF: ejection fraction, GFR: glomerular filtration rate, HDL: high-density lipoprotein, HT: hypertension, LA: left atrium, LDL: low-density lipoprotein, TIA: transient ischemic attack, TSH: thyroid-stimulating hormone, WBC: white blood cell. Descriptive statistics for continuous variables are shown as mean ± SD or median (25th–75th) percentiles, where appropriate. ^A^ Student’s *t* test, ^B^ Pearson’s χ^2^ test, ^C^ Mann–Whitney U test.

**Table 3:** Multivariable logistic regression analyses for independent predictors of severe stroke (NIHSS ≥ 15).

Variables	Multivariate Analysis–Model 1 ^a^		Multivariate Analysis–Model 2 ^b^	
	OR (95% CI)	*p*-Value	OR (95% CI)	*p*-Value
Carotid artery disease	2.190 (1.078–4.447)	0.030	2.552 (1.268–5.135)	0.009
Atrial fibrillation	1.488 (1.215–1.741)	0.025	1.578 (1.273–1.863)	0.021
ATRIA score	1.823 (1.568–2.119)	<0.001	–	–
Intermediate ATRIA (=6)	–	–	1.623 (0.616–4.277)	0.327
High ATRIA (>6)	–	–	11.692 (5.636–24.255)	<0.001
EF (%)	0.964 (0.932–0.997)	0.031	0.958 (0.928–0.989)	0.008
HbA1c (%)	1.072 (0.931–1.234)	0.334	1.052 (0.917–1.206)	0.471
CRP (mg/L)	1.009 (1.001–1.017)	0.021	1.009 (1.001–1.016)	0.025
Hemoglobin (g/dL)	0.988 (0.848–1.152)	0.880	0.997 (0.859–1.157)	0.966
Albumin (g/L)	0.970 (0.906–1.038)	0.380	0.968 (0.906–1.034)	0.334

CI: confidence interval, OR: odds ratio, EF: ejection fraction, CRP: C-reactive protein. ^a^ ATRIA score is presented as a continuous variable ^b^ ATRIA score is presented as a categorical variable; reference category for ATRIA score: <6.

## Data Availability

The data presented in this study are available upon request from the corresponding author. The data are not publicly available due to privacy and ethical restrictions.

## Supplementary Materials

The following supporting information can be downloaded at https://www.mdpi.com/article/10.3390/jcm14134665/s1, Table S1: ATRIA Score Components and Point Allocation Adapted from: ATRIA Stroke Risk Model; Table S2: The distribution of ATRIA scores in terms of stroke etiologies.

## References

[1] Mukherjee D., Patil C.G. (2011). Epidemiology and the global burden of stroke. World Neurosurg..

[2] Adams H., Davis P., Leira E., Chang K.-C., Bendixen B., Clarke W., Woolson R., Hansen M.D. (1999). Baseline NIH Stroke Scale score strongly predicts outcome after stroke: A report of the Trial of Org 10172 in Acute Stroke Treatment (TOAST). Neurology.

[3] Wolf P.A., Abbott R.D., Kannel W.B. (1991). Atrial fibrillation as an independent risk factor for stroke: The Framingham Study. Stroke.

[4] Benjamin E.J., Wolf P.A., D’Agostino R.B., Silbershatz H., Kannel W.B., Levy D. (1998). Impact of atrial fibrillation on the risk of death: The Framingham Heart Study. Circulation.

[5] Lin H.-J., Wolf P.A., Kelly-Hayes M., Beiser A.S., Kase C.S., Benjamin E.J., D’Agostino R.B. (1996). Stroke severity in atrial fibrillation: The Framingham Study. Stroke.

[6] Gage B.F., Waterman A.D., Shannon W., Boechler M., Rich M.W., Radford M.J. (2001). Validation of clinical classification schemes for predicting stroke: Results from the National Registry of Atrial Fibrillation. JAMA.

[7] Lip G.Y., Nieuwlaat R., Pisters R., Lane D.A., Crijns H.J. (2010). Refining clinical risk stratification for predicting stroke and thromboembolism in atrial fibrillation using a novel risk factor-based approach: The euro heart survey on atrial fibrillation. Chest.

[8] Mitchell L.B., Southern D.A., Galbraith D., Ghali W.A., Knudtson M., Wilton S.B. (2014). Prediction of stroke or TIA in patients without atrial fibrillation using CHADS_2_ and CHA_2_DS_2_-VASc scores. Heart.

[9] Hong H., Kim Y., Cha M.J., Kim J., Lee D., Lee H., Nam C., Nam H., Heo J. (2012). Early neurological outcomes according to CHADS_2_ score in stroke patients with non-valvular atrial fibrillation. Eur. J. Neurol..

[10] Tanaka K., Yamada T., Torii T., Furuta K., Matsumoto S., Yoshimura T., Takase K.-I., Wakata Y., Nakashima N., Kira J.-I. (2015). Pre-admission CHADS_2_, CHA_2_DS_2_-VASc, and R_2_CHADS_2_ scores on severity and functional outcome in acute ischemic stroke with atrial fibrillation. J. Stroke Cerebrovasc. Dis..

[11] Tu H.T., Campbell B.C., Meretoja A., Churilov L., Lees K.R., Donnan G.A., Davis S.M., Collaborators V. (2013). Pre-stroke CHADS_2_ and CHA_2_DS_2_-VASc scores are useful in stratifying three-month outcomes in patients with and without atrial fibrillation. Cerebrovasc. Dis..

[12] Henriksson K.M., Farahmand B., Johansson S., Åsberg S., Terént A., Edvardsson N. (2010). Survival after stroke—The impact of CHADS_2_ score and atrial fibrillation. Int. J. Cardiol..

[13] Sato S., Yazawa Y., Itabashi R., Tsukita K., Fujiwara S., Furui E. (2011). Pre-admission CHADS_2_ score is related to severity and outcome of stroke. J. Neurol. Sci..

[14] Singer D.E., Chang Y., Borowsky L.H., Fang M.C., Pomernacki N.K., Udaltsova N., Reynolds K., Go A.S. (2013). A new risk scheme to predict ischemic stroke and other thromboembolism in atrial fibrillation: The ATRIA study stroke risk score. J. Am. Heart Assoc..

[15] Brott T., Adams H.P., Olinger C.P., Marler J.R., Barsan W.G., Biller J., Spilker J., Holleran R., Eberle R., Hertzberg V. (1989). Measurements of acute cerebral infarction: A clinical examination scale. Stroke.

[16] Buleu F., Popa D., Williams C., Tudor A., Sutoi D., Trebuian C., Ioan C.C., Iancu A., Cozma G., Marin A.-M. (2024). Code Stroke Alert: Focus on Emergency Department Time Targets and Impact on Door-to-Needle Time across Day and Night Shifts. J. Pers. Med..

[17] Saver J.L., Goyal M., Van der Lugt A., Menon B.K., Majoie C.B., Dippel D.W., Campbell B.C., Nogueira R.G., Demchuk A.M., Tomasello A. (2016). Time to treatment with endovascular thrombectomy and outcomes from ischemic stroke: A meta-analysis. JAMA.

[18] Fonarow G.C., Saver J.L., Smith E.E., Broderick J.P., Kleindorfer D.O., Sacco R.L., Pan W., Olson D.M., Hernandez A.F., Peterson E.D. (2012). Relationship of national institutes of health stroke scale to 30-day mortality in medicare beneficiaries with acute ischemic stroke. J. Am. Heart Assoc..

[19] Smith E.E., Abdullah A.R., Petkovska I., Rosenthal E., Koroshetz W.J., Schwamm L.H. (2005). Poor outcomes in patients who do not receive intravenous tissue plasminogen activator because of mild or improving ischemic stroke. Stroke.

[20] Evans J.D. (1996). Straightforward Statistics for the Behavioral Sciences.

[21] Counsell C., Dennis M. (2001). Systematic review of prognostic models in patients with acute stroke. Cerebrovasc. Dis..

[22] Ntaios G., Lip G.Y., Makaritsis K., Papavasileiou V., Vemmou A., Koroboki E., Savvari P., Manios E., Milionis H., Vemmos K. (2013). CHADS_2_, CHA_2_DS_2_-VASc, and long-term stroke outcome in patients without atrial fibrillation. Neurology.

[23] Fang M.C., Go A.S., Chang Y., Borowsky L., Pomernacki N.K., Singer D.E., Group A.S. (2008). Comparison of risk stratification schemes to predict thromboembolism in people with nonvalvular atrial fibrillation. J. Am. Coll. Cardiol..

[24] Kim H.J., Park M.-S., Yoo J., Kim Y.D., Park H., Kim B.M., Bang O.Y., Kim H.C., Han E., Kim D.J. (2022). Association between CHADS_2_, CHA_2_DS_2_-VASc, ATRIA, and Essen Stroke Risk Scores and Functional Outcomes in Acute Ischemic Stroke Patients Who Received Endovascular Thrombectomy. J. Clin. Med..

[25] Sohrabji F., Bake S., Lewis D.K. (2013). Age-related changes in brain support cells: Implications for stroke severity. Neurochem. Int..

[26] Yahalom G., Schwartz R., Schwammenthal Y., Merzeliak O., Toashi M., Orion D., Sela B.-A., Tanne D. (2009). Chronic kidney disease and clinical outcome in patients with acute stroke. Stroke.

[27] Pantoni L. (2010). Cerebral small vessel disease: From pathogenesis and clinical characteristics to therapeutic challenges. Lancet Neurol..

[28] Kelly D.M., Pinheiro A.A., Koini M., Anderson C.D., Aparicio H., Hofer E., Kern D., Blacker D., DeCarli C., Hwang S.-J. (2024). Impaired kidney function, cerebral small vessel disease and cognitive disorders: The Framingham Heart Study. Nephrol. Dial. Transplant..

[29] Liu J., Rutten-Jacobs L., Liu M., Markus H.S., Traylor M. (2018). Causal impact of type 2 diabetes mellitus on cerebral small vessel disease. Stroke.

[30] Ormstad H., Aass H.C.D., Lund-Sørensen N., Amthor K.-F., Sandvik L. (2011). Serum levels of cytokines and C-reactive protein in acute ischemic stroke patients, and their relationship to stroke lateralization, type, and infarct volume. J. Neurol..

[31] Nakajima M., Kimura K., Ogata T., Takada T., Uchino M., Minematsu K. (2004). Relationships between angiographic findings and National Institutes of Health stroke scale score in cases of hyperacute carotid ischemic stroke. Am. J. Neuroradiol..

[32] Gorelick P.B., Wong K.S., Bae H.-J., Pandey D.K. (2008). Large artery intracranial occlusive disease: A large worldwide burden but a relatively neglected frontier. Stroke.

[33] Ratchford E.V., Evans N.S. (2014). Carotid artery disease. Vasc. Med..

[34] Buscemi S., Geraci G., Massenti F., Buscemi C., Costa F., D’Orio C., Rosafio G., Maniaci V., Parrinello G. (2017). Renal function and carotid atherosclerosis in adults with no known kidney disease. Nutr. Metab. Cardiovasc. Dis..

[35] Lima F.O., Furie K.L., Silva G.S., Lev M.H., Camargo É.C., Singhal A.B., Harris G.J., Halpern E.F., Koroshetz W.J., Smith W.S. (2010). The pattern of leptomeningeal collaterals on CT angiography is a strong predictor of long-term functional outcome in stroke patients with large vessel intracranial occlusion. Stroke.

[36] Hardigan T., Ward R., Ergul A. (2016). Cerebrovascular complications of diabetes: Focus on cognitive dysfunction. Clin. Sci..

[37] Yaghi S., Kamel H., Elkind M.S. (2017). Atrial cardiopathy: A mechanism of cryptogenic stroke. Expert Rev. Cardiovasc. Ther..

[38] Yaghi S., Boehme A.K., Hazan R., Hod E.A., Canaan A., Andrews H.F., Kamel H., Marshall R.S., Elkind M.S. (2016). Atrial cardiopathy and cryptogenic stroke: A cross-sectional pilot study. J. Stroke Cerebrovasc. Dis..

[39] Wu Y., Yang X., Jing J., Meng X., Li Z., Pan Y., Jiang Y., Yan H., Huang X., Liu L. (2023). Prognostic significance of atrial cardiopathy in patients with acute ischemic stroke. Eur. Stroke J..

[40] Chung M.K., Eckhardt L.L., Chen L.Y., Ahmed H.M., Gopinathannair R., Joglar J.A., Noseworthy P.A., Pack Q.R., Sanders P., Trulock K.M. (2020). Lifestyle and risk factor modification for reduction of atrial fibrillation: A scientific statement from the American Heart Association. Circulation.

