# Supplementary material for: Association Between Pre-Admission ATRIA Scores and Initial Stroke Severity in Acute Ischemic Stroke: A Cross-Sectional Study
Source: J Clin Med. 2025 Jul 1;14(13):4665. doi: 10.3390/jcm14134665 (PMC12250339; doi:10.3390/jcm14134665)
Supplement: Supplementary file 1 [file jcm-14-04665-s001.zip › jcm-3708194-supplementary.pdf]

Supplementary Table S1. ATRIA Score Components and Point Allocation Adapted from: ATRIA Stroke Risk Model

Component	Without a prior stroke	Prior stroke
Age		
≥85	6	9
75-84	5	7
65-74	3	7
<65	0	8
Female	1	1
Diabetes mellitus	1	1
Congestive heart failure	1	1
Hypertension	1	1
Proteinuria	1	1
Renal dysfunction (eGFR <45) or ESRD	1	1

Possible point scores range from 0 to 12 for those without a prior stroke and from 7 to 15 for those with a prior stroke. ATRIA: Anticoagulation and Risk Factors in Atrial Fibrillation, eGFR: estimated glomerular filtration rate, ESRD: end-stage renal disease.

Supplementary Table S2. The distribution of ATRIA scores in terms of stroke etiologies

	Cryptogenic	Carotid artery disease	Atrial fibrillation
**ATRIA category**			
Low (0-5)	69 (48.2%)[A]	52 (42.3%)[B]	43 (28.7%)[A,B]
Medium (6)	24 (16.8%)	20 (16.2%)	29 (19.3%)
High (7-15)	50 (35.0%)[A]	51 (41.5%)	78 (52.0%)[A]

[A] Cryptogenic *vs* Atrial fibrillation ($p<0.01$), [B] Carotid artery disease *vs* Atrial fibrillation ($p=0.019$).
